# Planktonic Aggregates of *Staphylococcus aureus* Protect against Common Antibiotics

**DOI:** 10.1371/journal.pone.0041075

**Published:** 2012-07-18

**Authors:** Jakob Haaber, Marianne Thorup Cohn, Dorte Frees, Thorbjørn Joest Andersen, Hanne Ingmer

**Affiliations:** 1 Department of Veterinary Disease Biology, University of Copenhagen, Frederiksberg, Denmark; 2 Department of Geography and Geology, University of Copenhagen, Copenhagen, Denmark; National Institutes of Health, United States of America

## Abstract

Bacterial cells are mostly studied during planktonic growth although in their natural habitats they are often found in communities such as biofilms with dramatically different physiological properties. We have examined another type of community namely cellular aggregates observed in strains of the human pathogen *Staphylococcus aureus*. By laser-diffraction particle–size analysis (LDA) we show, for strains forming visible aggregates, that the aggregation starts already in the early exponential growth phase and proceeds until post-exponential phase where more than 90% of the population is part of the aggregate community. Similar to some types of biofilm, the structural component of *S. aureus* aggregates is the polysaccharide intercellular adhesin (PIA). Importantly, PIA production correlates with the level of aggregation whether altered through mutations or exposure to sub-inhibitory concentrations of selected antibiotics. While some properties of aggregates resemble those of biofilms including increased mutation frequency and survival during antibiotic treatment, aggregated cells displayed higher metabolic activity than planktonic cells or cells in biofilm. Thus, our data indicate that the properties of cells in aggregates differ in some aspects from those in biofilms. It is generally accepted that the biofilm life style protects pathogens against antibiotics and the hostile environment of the host. We speculate that in aggregate communities *S. aureus* increases its tolerance to hazardous environments and that the combination of a biofilm-like environment with mobility has substantial practical and clinical importance.

## Introduction

Many bacterial species can grow either in the form of dispersed single cells in liquid or as densely packed communities attached to solid surfaces. Researchers generally refer to the latter growth form as biofilm, which was defined by Costerton as “a structured community of cells enclosed in a self-produced polymeric matrix and adherent to an inert or living surface” [1]. The opportunistic, human pathogen, *Staphylococcus aureus* can establish itself in biofilms by colonizing natural body surfaces including lungs and heart valves as well as abiotic surfaces such as medical implants [2], [3]. As cells present in biofilms are commonly protected against antibiotics and host defense molecules, biofilm formation has serious clinical consequences and is a significant contributor to the health care problems associated with *S. aureus* [4]–[6].

Staphylococcal biofilms contain several matrix components including extracellular DNA (eDNA), protein and polysaccharide [2], [7], [8]. In *S. aureus,* eDNA is released from dead cells by controlled cell lysis and the presence of eDNA is important in the very early establishment of the biofilm [9], [10]. At this early stage, *S. aureus* cell surface proteins such as fibronectin and fibrinogen binding proteins and Protein A are also contributing [11], [12]. The extracellular polysaccharide poly-N-acetyl-1,6-glucosamine (PNAG) is often involved in biofilm formation and it is the most characterized component of the biofilm matrix. PNAG is synthesized by the products of the polysaccharide intercellular adhesin (PIA) locus, *icaADBC* [13] and mediates adhesion to both living and artificial surfaces [14], [15]. The *ica* operon is present in many clinical *S. aureus* strains and its expression has been shown to be strongly induced in a device-related animal model, underscoring the importance of biofilms during infection [16].

During establishment of an infection, formation of biofilm may be seen as a survival strategy against host defenses and antimicrobial therapy. It is well accepted that biofilms protect the embedded cells against antimicrobial therapy [17]–[20] for example through reduced exposure to the antimicrobial compounds [21], [22] or reduced metabolic activity that decreases susceptibility to a range of antibiotics [20]. However, although a biofilm can provide beneficial properties to the participating bacteria it also traps the cells in a sessile community with limited mobility. This limitation seems to have been circumvented by the human, opportunistic pathogen, *Pseudomonas aeruginosa* that upon growth in liquid culture forms large aggregates containing densely packed viable cells and eDNA [23]. The properties of *P. aeruginosa* cells present in aggregates resemble those of flow-cell biofilms by their slow growth rate, their capacity to survive otherwise lethal treatments with antibiotics and their ability to resist phagocytosis [24]. Interestingly, non-attached aggregates are also observed in the lungs of cystic fibrosis patients, in soft tissue fillers and in non-healing wounds, indicating that they are of clinical relevance [24]–[26].

Planktonic (non-attached) aggregation has also been observed for other bacterial species. In the food borne pathogen, *Campylobacter jejuni,* auto-aggregation is regularly observed but dispersal is promoted in mutants with disrupted stress response pathways [27], [28]. *Streptococcus pyogenes* is another human pathogen that forms planktonic aggregates in liquid culture and this property is important for adherence, resistance to phagocytosis and virulence [29]. *S. aureus* is known to grow in small clusters of 5–20 cells but it also assembles in large aggregates that are visible to the naked eye and are observed in both laboratory and clinical settings [12], [26]. While extensive research has focused on staphylococcal biofilms only little knowledge exists on the formation and properties of *S. aureus* planktonic aggregation.

Here, we characterize *S. aureus* aggregation and show that while aggregates share many of the properties of surface-bound biofilms, differences are also observed. In common to biofilms, the aggregation process is influenced by sub-lethal concentrations of antibiotics [30] and cells in aggregates are protected against several unrelated and clinically relevant antimicrobial compounds [19], [20]. In contrast to biofilms, however, metabolic activity is high in aggregates. We speculate that aggregates provide bacteria with the benefits of a biofilm while maintaining mobility and that this combination may contribute to the difficulties of eradicating *S. aureus* infections.

## Results

### *Staphylococcus Aureus* Aggregates in Solution

**Figure 1:**
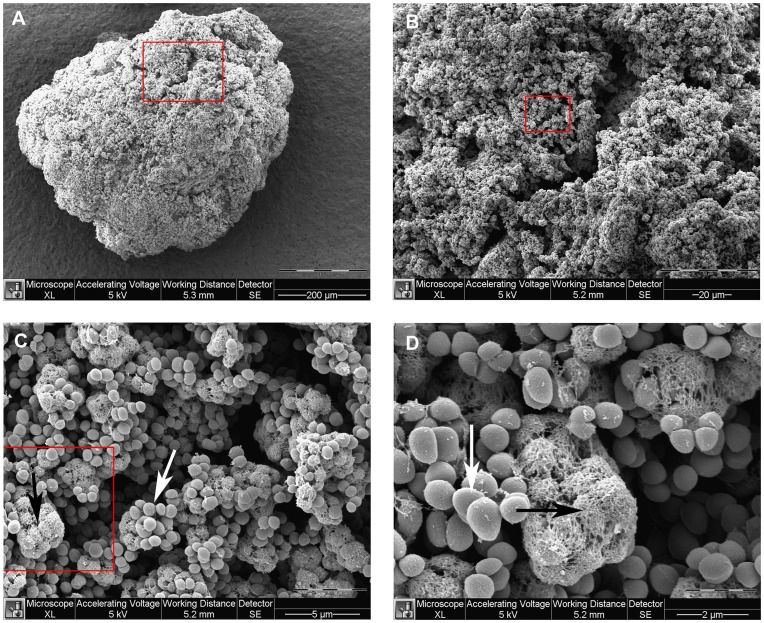
Aggregate visualized by SEM. Aggregates from a post-exponential 8325-4 culture were fixed and visualized using Scanning Electron Microscopy (SEM). Panels A-D represent increasing magnification and red squares indicate the area magnified in the following panel. Overview of an aggregate, which is visible to the naked eye (A). Zooming in reveals pores and crevices in a topographical landscape of aggregated clusters (B). At higher magnifications it is revealed that some clusters are embedded in a web-like matrix (black arrows) while some are not (white arrows) (C, D).

Some strains of *S. aureus* are capable of forming large planktonic aggregates that are visible to the naked eye when grown under standard laboratory conditions [11], [12], [31]. From previous unpublished work we had noticed four strains with very different aggregation capabilities. These were two clinical isolates (15981 and SA564) and two laboratory strains (8325-4 and Newman), where 8325-4 and 15981 form visible planktonic aggregates, while Newman and SA564 do not (data not shown). With the aim of investigating this phenomenon in more detail, we applied scanning electron microscopy (SEM) to visualize aggregates in a post-exponential culture of 8325-4 (Figure 1). Some aggregates were more than 0.5 mm of size and consisted of a large numbers of cells (Figure 1A). A closer look revealed pores and crevices in the compact mass of cells (Figure 1B) and at even higher magnification some cell-clusters were covered in a web-like structure as recently observed in biofilms of *P. aeruginosa* [32] while other clusters of cells remained un-covered (Figure 1C, D).

**Figure 2:**
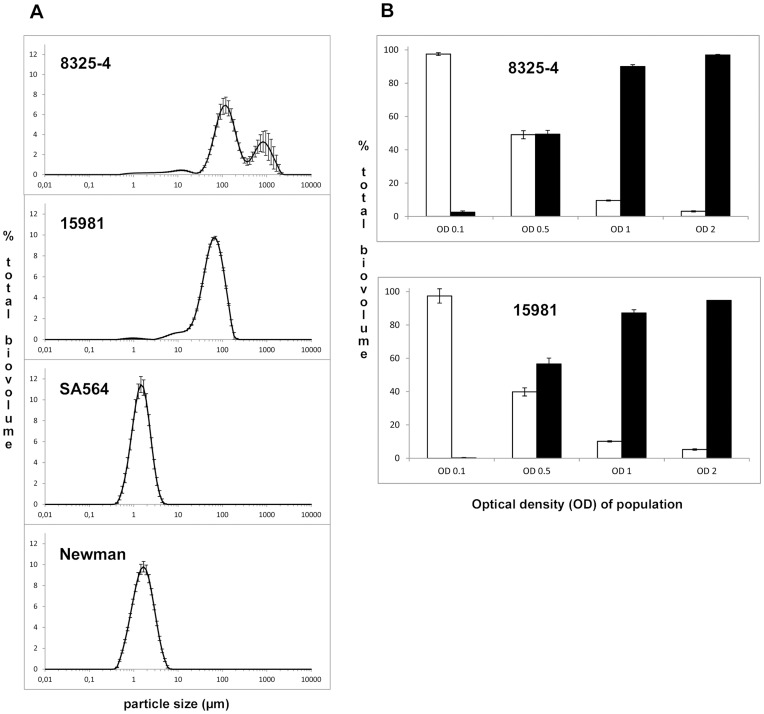
Aggregate size distribution measured by LDA. Four different *S. aureus* strains were investigated for their level of aggregation in post-exponential growth phase (OD_600_ = 2) (A). The two aggregating strains were followed through exponential growth and the percentage of cells in aggregates (>6 µm, solid bars) relative to the non-aggregating fraction (open bars) is shown (B). % total biovolume is calculated as the percentage of cells of a given size relative to the total suspended cell mass. Error bars indicate standard deviation (n = 5).

To quantify the extent of aggregation in liquid cultures of the four *S. aureus* strains, we monitored the formation of planktonic *S. aureus* aggregates using laser diffraction analysis (LDA) [23]. When grown to post exponential growth phase (OD_600_ = 2) the majority of cells of strains 8325-4 and 15981 were assembled in particles of more than 6 µm in diameter, the minimum size chosen to define an aggregate. For strains Newman and SA564 the average particle size was 1–2 µm in diameter (Figure 2A) representing single and double cells as observed by microscopy. Further, the LDA analysis revealed that aggregate size varies between strains. For 8325-4 cultures two aggregate sizes of 100 and 800 µm in diameter predominate while for strain 15981 the aggregates are approximate 60 µm in diameter (Figure 2A).

To address the structural robustness of the *S. aureus* aggregates we applied increasing shear force as part of the LDA and found that with gradual steps of stirring (50–500 rpm) and pump speed (250–2500) no changes were observed in the size distribution until medium shear force was applied (stir = 300 rpm, pump = 1500 rpm). Even when exposed to maximum shear (stir = 500 rpm, pump = 2500 rpm), the aggregates were still surprisingly intact with a reduction in average aggregate size from 180 µm to 45 µm and maximum size from 480 µm to 240 µm (Figure S1). Thus, under the assayed conditions the *S. aureus* aggregates are structurally stable, which is in contrast to aggregates formed by *P. aeruginosa* that are readily dissolved by whirly mixing [24].

### Aggregates are Formed already in the Exponential Phase

To address the temporal development of aggregate formation we monitored particle size of strains 8325-4 and 15981 at various growth stages and found that while very little aggregation occurred at OD_600_ of 0.1, already at OD_600_ of 0.5 around 50% of the cells were assembled into aggregates (Figure 2B). Thus, for the tested *S. aureus* strains, aggregation is not confined to the post-exponential growth phase but occurs already at mid-exponential phase. The size of the aggregates was two to three times larger for 8325-4 than for 15981. Interestingly though, when applying the 6 µm threshold for defining aggregates, the distribution of cells in the aggregated versus dispersed fractions of cells was strikingly similar for strains 8325-4 and 15981 throughout the growth cycle (Figure 2B).

**Figure 3:**
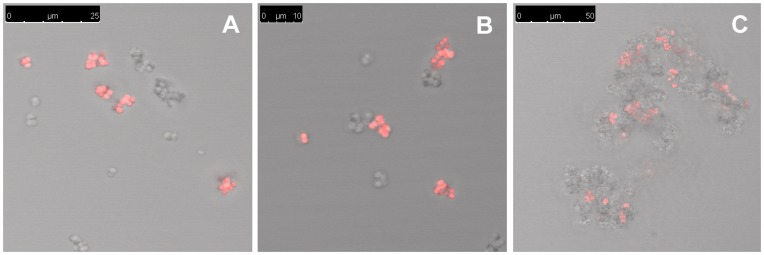
Kinetic of aggregate formation. 8325-4 cells were mixed 1∶1 with 8325-4 cells expressing YFP to OD_600_ of 0.01 and examined using CLSM every 30 min through a growth cycle. Small clusters of cells dominate until OD_600_ = 0.1 (A) at which time they start to fuse (B) and form large aggregates around OD_600_ = 0.5 (C). Note different sizes of scale bars.

To address whether aggregates are formed by interconnected daughter cells that are not separated after cell division or from aggregation of single cells or smaller clusters we monitored aggregation of fluorescent and non-fluorescent, isogenic 8325-4 cells after mixing (1∶1) at OD_600_ = 0.01 [33]. The results show that in the early growth stages (OD_600_ = 0.01 to 0.1) small grape-like clusters are formed by ≈10 interconnected daughter cells (Figure 3A). At OD_600_ = 0.1 the clusters of different clonal origin begin to merge (Figure 3B) and from OD_600_ = 0.5 an increasing number of large aggregates are observed that are composed of both labeled and non-labeled cells (Figure 3C). Thus, our results suggest that the large multi-cellular aggregates observed in post-exponential growth phase arise from small cell clusters that merge during exponential growth phase and end up glued together in large multi-cellular structures.

### Sub-inhibitory Concentrations of Antibiotics and Environmental Factors Influence Aggregation

**Figure 4:**
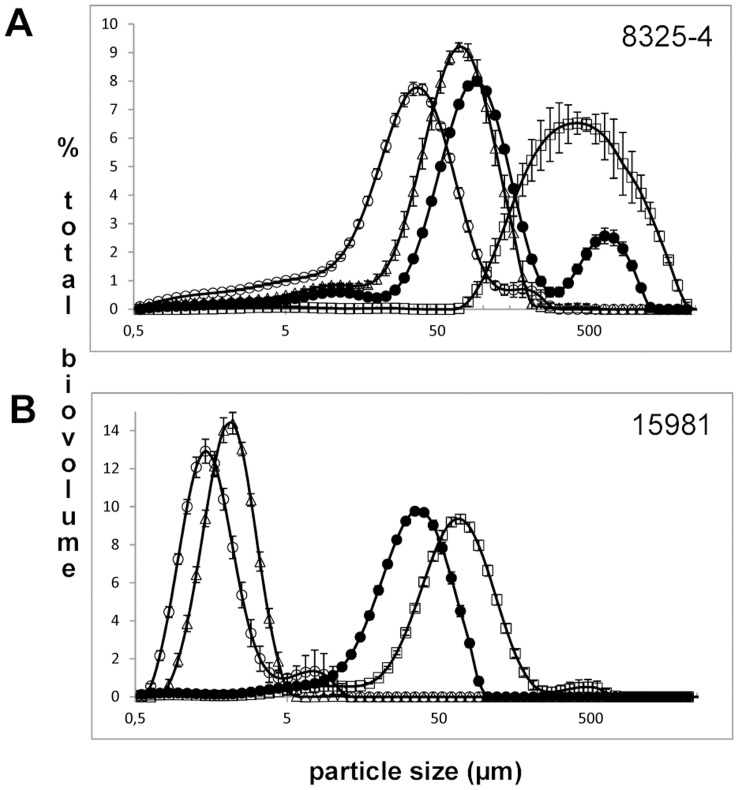
Aggregation is influenced by the presence of sub-inhibitory concentrations of antibiotics. 8325-4 (panel A) and 15981 (panel B) were cultivated to OD_600_ of 2 in TSB (solid circle) or in TSB added 1/25×MIC of erythromycin (open triangles), cefuroxime (open circles) or rifampicin (open squares). The size distribution of planktonic aggregates was examined using LDA. Error bars indicate standard deviation (n = 5).

Bacterial pathogens may be exposed to sub-lethal concentrations of antibiotics as a consequence of uneven antibiotic distribution during antimicrobial therapy, inadequate use of antibiotics or from exposure to antimicrobials naturally produced in soil and water environments [34]–[36]. Notably, we observed that addition of sub-inhibitory concentrations (1/25 × MIC) of several clinically relevant antibiotics influenced the aggregation properties of the aggregating strains 8325-4 and 15981 (Figure 4). The RNA polymerase inhibitor rifampicin stimulated aggregation while the protein biosynthesis inhibitor erythromycin and the cell wall synthesis inhibitor cefuroxime reduced aggregation. Further we examined the aggregation ability in 43 *S. aureus* strains from our culture collection comprising laboratory strains and clinical isolates. We found that while significant differences were observed between strains, exposure to either 2% NaCl, 35 mM glucose or reduced oxygen tension induced aggregation in all strains tested (data not shown) suggesting that the ability to aggregate is omnipresent in *S. aureus*.

### The Polysaccharide Intercellular Adhesion (PIA) Promotes Aggregate Formation

The cellular level of the polysaccharide intercellular adhesin (PIA) produced by the enzymes encoded by the *icaADBC* operon has been noted to influence dispersed growth of staphylococci in liquid cultures [31], [37]. To evaluate the contribution of PIA to aggregation in *S. aureus*, we examined aggregate formation in an isogenic *ica* mutant of 8325-4 during post-exponential growth. No visible aggregates were observed and using LDA we could verify that no particles larger than 6 µm were present in the culture demonstrating that aggregates are not formed in the absence of PIA (Figure S2). In an isogenic *ica* mutant of 15981 aggregation was also abolished (data not shown).

**Figure 5:**
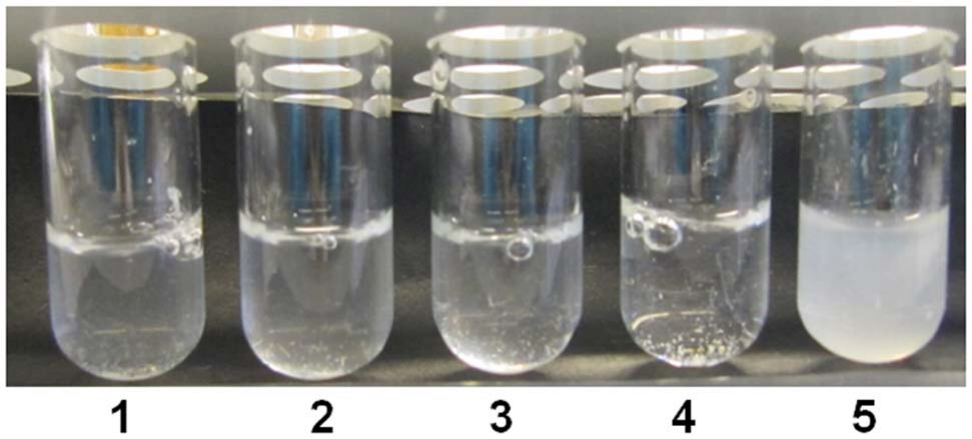
Polysaccharide constitutes the extracellular matrix of 8325-4 aggregates. Aggregates of 8325-4 cells were treated with DNase (tube 2), proteinase K (tube 3) or sodium metaperiodate (tube 5) at 37°C for 18 hours. Tubes 1 and 4 are untreated controls.

To further investigate the composition of the extracellular matrix in *S. aureus* aggregates we examined the resistance to the action of either proteinase K, DNase or metaperiodate, which degrade protein, DNA and polysaccharide, respectively. Only the addition of metaperiodate resulted in disruption of aggregates (Figure 5). This result indicates that extracellular polysaccharides form the major adhesive component of the aggregates thus supporting the genetic data stating the importance of the *ica* operon.

**Figure 6:**
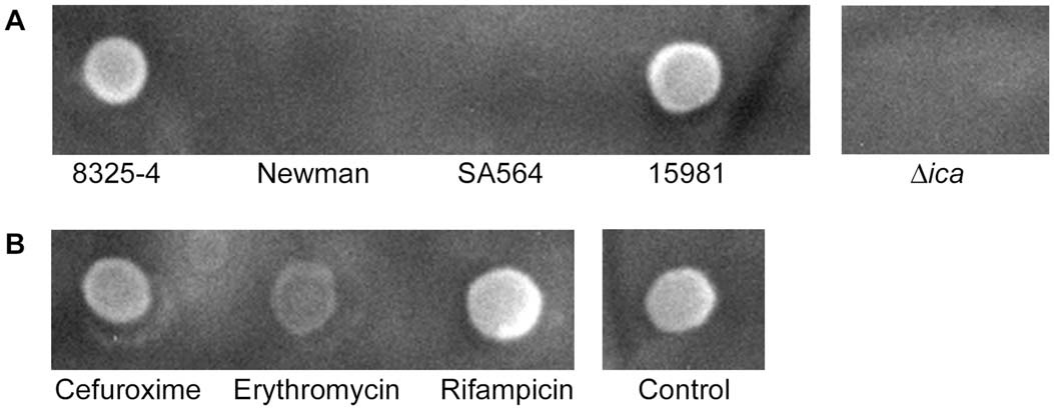
Production of the Polysaccharide Intercellular Adhesin (PIA). Dot blot and immune-detection was used to determine the amount of PIA produced by different strains in post-exponential growth phase (A) or by strain 8325-4 after exposure to 1/25 MIC of different antibiotics (B).

The central role of PIA polysaccharide was further confirmed by serological detection of PIA production in aggregating and non-aggregating strains. Here we observed a clear correlation between PIA production and aggregation (Figure 6A). Furthermore, analysis of the PIA level in 8325-4 cultures treated with antibiotics showed that rifampicin increased the cellular PIA level, while cefuroxime and erythromycin decreased PIA production (Figure 6B). The PIA levels correlated well with the level of aggregation of 8325-4 treated with these antibiotics (Figure 4). Collectively, these results indicate that the production of PIA is a major contributor to planktonic aggregate formation of *S. aureus* and that sub-inhibitory concentrations of antibiotics may affect aggregation, possibly through modulation of PIA production.

In addition to PIA other cellular components such as the fibronectin binding proteins have been reported to influence biofilm formation [11]. However inactivation of *fnbpA, fnbpB* and *fnbpAB* in strain 8325-4 did not influence particle size when compared to wild type cells suggesting that these proteins do not contribute to *S. aureus* aggregation (Figure S2). In addition we examined aggregation in a mutant strain of 8325-4 lacking *agr*, the *S. aureus* quorum sensing locus that controls virulence gene expression and in SH1000, a Sigma B (*rsbU^+^*) complemented strain of 8325-4. While both mutant strains are reported to be good biofilm formers [15], [25] they were essentially unable to aggregate (Figure S2). Thus, our data suggest that some elements of aggregation differ from biofilm formation and that the processes behind may not be identical.

### Aggregation Increases Metabolic Activity and Mutation Frequency

**Figure 7:**
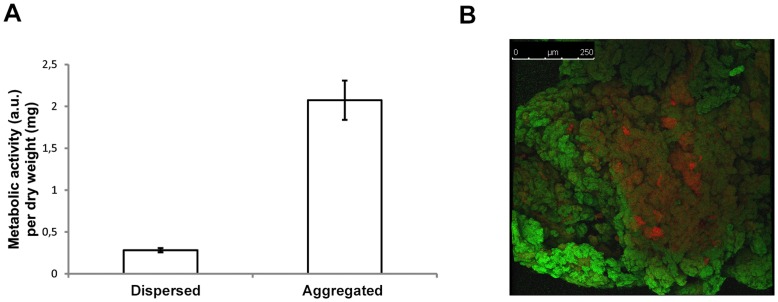
Metabolic activity is significantly higher in aggregated compared to dispersed cells. Metabolic activity was determined in a post-exponential (OD_600_ = 2) culture of 8325-4 by measuring reduced XTT (arbitrary units) normalized to mg dry weight (A). Error bars indicate standard deviation (n = 3). The distribution of active cells (green) and cells with low membrane potential (red) in aggregates was determined using LIVE/DEAD staining and CLSM (B).

Phenotypic properties of biofilm-associated cells vary from those of single cells in several ways including metabolic activity [6] and mutation frequency [38], [39]. We investigated whether this is also the case for aggregated cells. The metabolic activity of cells correlates with the reduction of a tetrazolium salt (XTT) to the colored compound formazan [40], [41]. When applying this colorimetric method to evaluate the metabolic activity of aggregated and planktonic cells, we surprisingly found that on average the metabolic activity of cells in aggregates was approximately 7 fold higher than in planktonic cells (p<0.001, Figure 7A). This is in contrast to what has been reported for biofilm associated cells, where oxygen and nutrient limitation results in low metabolic activity [20]. Accordingly, we saw approximately 4 fold lower metabolic activity in a biofilm compared to dispersed cells (p<0.001) (Figure S3). When comparing metabolic activity of aggregates with cells originating from aggregates disrupted by sonication, no differences were observed (data not shown, p = 0.84). Furthermore, when comparing isogenic *ica* mutants of both 8325-4 and 15981 to aggregates and dispersed cells, metabolic activity was significantly higher in the aggregates than in dispersed wild type and *ica* mutant cells (Figure S3, p<0.05). In biofilms, the metabolic activity is unevenly distributed depending on oxygen and nutrient availability [42]. CLSM of LIVE/DEAD stained aggregates revealed that analogous to biofilms, aggregates are unevenly composed of areas of cells with low metabolic activity surrounded by metabolically active cells (Figure 7B). Collectively, our results show that on average cells in aggregates are markedly more metabolically active compared to those in biofilms or planktonic cells.

Mutation frequency has previously been reported to be higher in biofilms compared to single cells [38], [39]. We determined the mutation rate, calculated by the number of generated rifampicin-resistant-mutants per total CFU, and found that it was 2 fold greater in the aggregate fraction compared to the dispersed cell fraction (Figure S4, p = 0.0015). Thus, aggregates resemble biofilms by having a slightly increased mutation frequency compared to planktonic cells.

### Aggregation Promotes Protection against Antibiotic Killing

**Figure 8:**
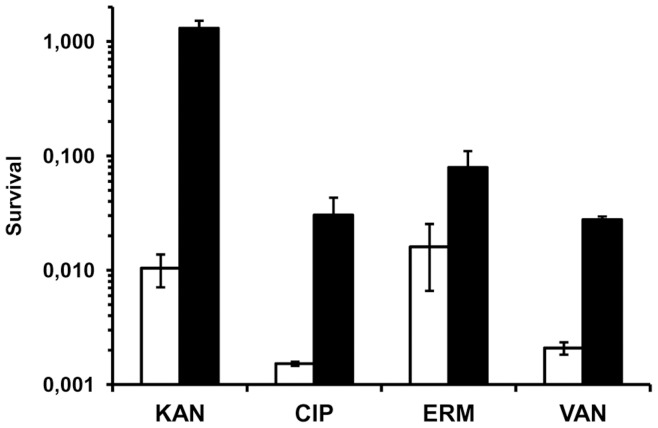
Aggregated cells have increased survival after antibiotic treatment. Cells in aggregates (solid bars) survive better than dispersed cells (open bars) following treatment with 25×MIC of kanamycin (KAN), ciprofloxacin (CIP), erythromycin (ERM) or vancomycin (VAN). Survival was calculated as CFU present in the aggregate and dispersed fractions after treatment with antibiotics relative to CFU measured before antibiotic exposure. Error bars indicate standard deviation (n = 3).

Interestingly, we noted that the biomass of dispersed cells increased during stationary phase compared to the aggregated fraction (data not shown) indicating that cells might detach from the aggregates to become dispersed as observed for *P. aeruginosa* when experiencing starvation [23]. Therefore, we used an experimental set up with antibiotic markers that enabled us to follow cells from the two fractions after antibiotic exposure. Aggregated and dispersed cells with different antibiotic markers were mixed and exposed to 25× MIC of clinically relevant antibiotics with different cellular targets (kanamycin, ciprofloxacin, erythromycin and vancomycin). Importantly, antibiotic exposure mediated increased shedding of cells from the aggregate fraction to the dispersed fraction (data not shown, p<0.05). Furthermore, we found that cells assembled in aggregates were protected against the lethality of antibiotics and survived significantly better (p<0.05) compared to dispersed cells (Figure 8). For kanamycin there was a dramatic (100×) increase of survival in the aggregated cells compared to dispersed cells, while for ciprofloxacin, vancomycin and erythromycin the increased survival was 30×, 14× and 8× higher, respectively (Figure 8). However, if aggregates were disrupted by sonication and the released cells were exposed to kanamycin, the protection was completely abolished and the susceptibility of the cells was comparable to the non-aggregated cell fraction (data not shown, p<0.01). This is equivalent to observations in *P. aeruginosa* in which tobramycin regained its efficacy after disruption of the aggregates [24] and it indicates that protection against antibiotics in aggregates in these two species is mediated by a physical barrier provided by the aggregate matrix as also observed for flocculating yeast [43].

## Discussion

The ability of *Staphylococcus aureus* to form biofilms on host tissues and medical implants is considered to be one of the most important traits contributing to the health care problems associated with the organism [11], [44], [45]. Recently it was proposed for another opportunistic pathogen, *P. aeruginosa,* that non-attached planktonic aggregates of cells may be considered dispersed biofilms and that these planktonic aggregates have the same protective properties as biofilms [24]. Sporadically, *S. aureus* has been reported to form planktonic aggregates under laboratory conditions [12], [31] and in clinical infections [26], [46] but so far this property has received little attention.

We show here that the ability of *S. aureus* to form large planktonic aggregates is highly dependent on strain and growth conditions and is observed for both clinical and laboratory strains. The aggregation process starts already in the early exponential growth phase. At low cell densities *S. aureus* 8325-4 grows in clonal, structured populations of up to approx. 20 cells whereas at greater cell densities these structures merge into large aggregates measuring up to 1000 µm in diameter.

The matrix component responsible for the extensive aggregation is a polysaccharide and is likely to be the extracellular polysaccharide PIA, as Western blot analysis correlated the level of aggregation to PIA production and absence of the *ica* operon (encoding the PIA producing enzymes) completely abolished aggregation. The conserved *ica* operon is present in most clinical isolates of *S. aureus* [45] and its expression is induced in exponential growth phase [16], [31] and during infection [16]. Furthermore, clinical data show that *S. aureus* form PIA embedded multi-cellular aggregates within the mucus of cystic fibrosis patients [45]. Taken together, PIA is likely to contribute to both the aggregation process and the pathogenesis of *S. aureus*.

Although little is still known about the environmental factors that promote aggregation, we observed that different environmental conditions (NaCl, glucose, O_2_) affected the aggregation ability of many *S. aureus* sub-species, indicating that aggregation may be a common feature of staphylococci. Interestingly, also sub-inhibitory concentrations of antibiotics affected aggregation. As the effects were correlated with corresponding changes in PIA production we propose that sub-inhibitory concentrations of antibiotics may affect aggregation of *S. aureus* by modulating the PIA production. This may also be the case for other staphylococcal species as antibiotics have been shown to stimulate *ica* expression in *Staphylococcus epidermidis* [47].

The central role of PIA in formation of aggregates and some types of biofilm indicated that the *S. aureus* aggregates may possess properties resembling biofilms. The initial steps involved in biofilm formation and aggregation, however, are likely to be different since fibronectin binding proteins A and B promote biofilm formation but do not contribute to aggregation. Furthermore, strong biofilm formers such as an *agr* mutant in 8325-4 and the *rsbU* ^+^ complemented SH1000 showed very limited aggregation potential. On the other hand, protein A encoded by *spa* has previously been shown to promote both biofilm and aggregation of *S. aureus* [12] and supportive of this, we observed that a *spa* mutation in strain 8325-4 abolished aggregation (results not shown). However, the existence of both PIA-dependent [37] and PIA-independent [48] biofilms may complicate the interpretations of these results. Taken altogether, our results suggest that formation of aggregates and biofilms may not be identical processes.

Biofilms are generally considered to have low metabolic activity [20] and accordingly, we found metabolic activity in surface attached biofilms to be significantly lower than dispersed planktonic cells. However, when we examined the metabolic activity of the non-attached aggregated cells, we unexpectedly found it to be 7–8 fold higher compared to dispersed cells. In biofilms, dead cells have previously been proposed as source a of nutrition for a growing surface layer of cells [48]. Confocal laser scanning microscopy of LIVE/DEAD stained aggregates indeed showed that in analogy to biofilms, cells with low membrane potential dominate the center of these aggregates while a layer of metabolically active cells coat the aggregates, which may explain the increased metabolic activity observed in the aggregated compared to the dispersed cell fraction. Isogenic *ica* mutants of 8325-4 and 15981 had low levels of metabolic activity, which was comparable to the dispersed cell fraction of the respective wild types emphasizing the significance of the aggregate life style for high metabolic activity. Furthermore, following sonication, cells from disrupted aggregates retained their high metabolic activity and collectively, these results suggest that cells in aggregates are experiencing more growth stimulating conditions than both dispersed and biofilm-dwelling cells.

Another significant hallmark of biofilms is their resistance to antimicrobial compounds [4], [6]. Importantly, we found that the *S. aureus* aggregates are tolerant to various antibiotics. The antibiotics have very different cellular targets (protein-, DNA- and cell wall synthesis) indicating that the mechanism conferring the tolerance to the aggregated cells probably is general. Several mechanisms have been suggested to explain the reduced killing of cells in biofilms including reduced penetration of antibiotics into the biofilm, decreased growth rate, persister cells and phenotypic variants [49]–[52]. In contrast to the aggregates formed by *P. aeruginosa* [24], the *S. aureus* aggregates are highly robust and resistant to substantial shear forces suggesting that the density of the matrix may offer protection against antimicrobial compounds. Indeed, we found that disruption of the aggregates by sonication completely abolished the protection against kanamycin. In accordance with observations in *P. aeruginosa* and *Saccharomyces cerevisiae* this indicates that protection against antibiotics is a consequence of a physical barrier provided by the aggregates rather than the physiological state of the cells [24], [43].

Social behavior is commonly observed for eukaryotic organisms where aggregation appears to be a highly controlled process that reflects adaptive behavior in response to adverse environmental conditions [43], [53]. Less is known about social behavior in prokaryotic organisms but it is an intriguing speculation that aggregation may be an adaptive response that allows *S. aureus* to withstand host defenses and antimicrobial therapy. The results in this study represent *in vitro* experiments and studies investigating the clinical impact of the aggregation phenotype are highly warranted. In conclusion, we have shown that *S. aureus* under laboratory conditions is capable of forming large aggregates as a consequence of enhanced production of the PIA polysaccharide. We demonstrate similarities to surface bound biofilms including an increased tolerance towards antibiotics, and we speculate that aggregates due to the biofilm-like properties combined with mobility may be of significant clinical importance.

## Materials and Methods

### Bacterial Strains and Culture Conditions

The bacterial strains and plasmids used in this study are listed in Table 1. Strains were grown in tryptic soy broth (TSB; Oxoid) or on tryptic soy agar (TSA; Oxoid). Under standard growth conditions cultures were grown in Erlenmeyer flasks with an air:TSB volume ratio of 10∶1. TSB was inoculated with cells from TSA plates to OD_600_ of 0.01 and incubated at 37°C with agitation (160 rpm) until visible aggregation occurred in late exponential phase (approx. 4 h growth). When appropriate, 2% NaCl, 35 mM glucose was supplemented to the TSB. For reduced O_2_ exposure, cultures were grown in tightly sealed Erlenmeyer flasks to prevent air exchange. Biofilm formation experiments were performed in TSB supplemented with 35 mM glucose in Erlenmeyer flasks incubated at 37°C with no agitation. If sonication was applied before plating, cells were suspended in 1 ml 0.9% NaCl or TSB and sonicated 15 pulses, 500 msec, 50% power using a Bandelin sonopuls HD2070/UW2070 (Bandelin electronics, Germany) apparatus. Large aggregates sedimented quickly when shaking was stopped and therefore care was taken to manually agitate the flasks to ensure an even distribution of aggregates in the flasks immediately before sampling. All cultures containing aggregates were handled using pipette tips with minimum 2.5 mm opening to prevent shearing of the aggregates.

**Table 1:** Strains and plasmids used in the study.

Strains and plasmids	Relevant characteristics	Source and reference
Strains		
8325-4	Wild type strain 8325 cured of phages φ11, φ12 and φ13	[55]
RN4220	Restriction-defective derivative of RN450	[56]
SH1000	Functional *rsbU* derivative of 8325-4 *rsbU* ^+^	[57]
15981	Clinical strain	[58]
Newman	Clinical isolate (ATCC 25904)	[59]
SA564	Low passage clinical isolate from a patient with toxic shock syndrome	[60]
ATC35556Δ*ica::tet*	Does not produce PNAG/PIA	[13]
8325-4Δ*ica::tet*	Transduced from ATC35556Δ*ica::tet*	This study
15981Δ*ica::tet*	Does not produce PNAG/PIA	[61]
DU5723	Protein A negative strain derivative of 8325-4	[63]
RN6911	*RN6390*Δ*agr::tet*. A 8325-4 derivative not expressing the *agr* quorum sensing locus	[62]
8325-4-pRMC2	8325-4 harbouring pRMC2	This study
8325-4-rifR	Spontaneous rifampicin resistant mutant of 8325-4	This study
8325-4-CFP	8325-4 harbouring PJEBAN2	This study
8325-4-YFP	8325-4 harbouring PJEBAN3	This study
Plasmids		
PJEBAN2	EmR, Mob+(IncP), *oriR* pAMβ1, *oriR* pUC; *Pdlt-cfp+*	[33]
PJEBAN3	EmR, Mob+(IncP), *oriR* pAMβ1, *oriR* pUC; *Pdlt-yfp+*	[33]
pRMC2	*cat*, *bla*; *tetR*,Pxyl/tet (1x*tetO*),*E. coli*/*Staphylococcus* shuttle vector, pALC2073 derivative	[64]

### LDA-particle Sizing of Aggregates in Liquid Cultures

Laser-diffraction analysis was performed essentially as reported previously [23] (Text S1). As our standard condition on the Malvern Mastersizer 2000 instrument we used a low stirring speed of 50 rpm and pump speed of 250 rpm, which kept aggregates in suspension and ensured an even flow of aggregates into the detection chamber while still keeping shear forces at a minimum. As the cell populations of both of the non-aggregating strains (Newman and SA564) were in the size fraction <6 µm, we used this value as the cut-off to distinguish between dispersed cell and aggregate fractions.

### Procedure for Separation of Aggregates from Dispersed Cells

Ten mL culture was aliquoted into a 12 mL Falcon tube and aggregates were allowed to sediment for 10 min before a quick spin of 1400 rpm, 15 s was applied. The separation procedure is a compromise since it was not possible to pellet all aggregates while keeping dispersed cells in suspension. Therefore, the length and speed of the quick spin step was calibrated to yield no visible cell pellet using a fully dispersed culture of a non-aggregating strain but a visible pellet when applied to an aggregating culture. When analyzing the supernatant after the quick spin procedure of such a culture it was revealed that all large aggregates (>50 µm) were removed and the majority of the cells were in the size fraction between single cells (1 µm) and 15 µm (Figure S5).

### Fluorescent Tagging of *S. aureus*

Standard *S. aureus* transformation procedure was used by first amplifying PJEBAN3 in RN4220 (R^−/^M^+^) before electroporating it into 8325-4 followed by selective plating on TSA plates containing 5 µg/ml erythromycin to obtain 8325-4 expressing yellow fluorescent protein (YFP). The same procedure was used to introduce PJEBAN2 into 8325-4 to obtain cells expressing the cyan fluorescent protein (CFP). However, the CFP signal was not detectable in this strain and thus the strain carrying PJEBAN2 was used as control in studies with mixed cultures.

### Microscopy of Aggregates

Prior to CLSM microscopy of non-fluorescent cellular aggregates cells were stained using LIVE/DEAD® *Bac*light™ Bacterial Viability Kit (Invitrogen) according to the manufacturers’ recommendations.

Confocal laser scanning microscopy was carried out using a Leica SP5-X confocal laser scanning microscope (CLSM), equipped with an argon and white light laser for excitation of the flourophores (excitation/emission): SYTO 9 (480 nm/500 nm), propidium iodide (490 nm/635 nm) and YFP (514 nm/565 nm). Images were obtained by overlaying either 2 fluorescent channels alone or in combination with a transmission (brightfield) channel. All images were generated and processed using the LAS AF version 2.3.5 (Leica Microsystems, Germany).

Scanning Electron Microscopy (SEM) was performed as previously described [54]. In short, aggregates were fixed in 2% glutaraldehyde and postfixed in 1% OsO_4_. Samples were then dehydrated in ethanol, critical point-dried using CO_2_, and sputter-coated with gold according to standard procedures. Samples were investigated using a Philips XL Feg30 scanning electron microscope operated at 5 kV accelerating tension.

### Degradation of Planktonic Aggregates

Aggregates were separated from dispersed cells before being resuspended in 500 µl 50 mM sodium acetate buffer (pH 4.5) and added 250 µl 10 mM sodium metaperiodate (Sigma) or in 500 µl Tris with 100 mM NaCl (pH 7.5) and added either 100 µl DNase 1 U/µl (Fermentas) or 250 µl 100 µg/ml proteinase K (Sigma). Samples were incubated at 37°C for 18 hours.

### Dot Blot Analysis of PIA Level

The PIA level in *S. aureus* strains was detected as described by Cramton *et al.,* 1999 with few modifications [13] (Text S1).

### Metabolic Activity

A colorimetric assay in which the colorless XTT (2,3-bis-(2-methoxy-4-nitro-5-sulfophenyl)-2H-tetrazolium-5-carboxanilide) (Sigma) is reduced to the water soluble formazan dye by metabolically active cells was used to compare the metabolic activity between biofilm cells, aggregated cells and dispersed cells basically as described before [40], [41]. Biofilm was scraped off the glass surface of Erlenmeyer flasks following two times rinsing with 0.01 M PBS (pH 7.4) and from liquid cultures, the dispersed cell and aggregate fractions were separated and harvested. From each of the three fractions 2-10 mg cell mass was resuspended in 500 µl of a filter-sterilized 0.5 mg/ml XTT and 50 µM menadione (sigma) solution. Following 3 h incubation (37°C, 160 rpm agitation) in the dark, the cells were harvested and reduction of XTT was measured in the supernatant at 492 nm using an eppendorf BioPhotometer Plus spectrophotometer. The pelleted cells were dried (55°C, 18 h) to determine the dry weight. A standard curve revealed a linear relationship between dry weight and XTT reduction from at least 1 to 13 mg of dry weight cell mass (data not shown).

### Mutation Frequency

Aggregating cultures were separated into aggregate and dispersed cell fractions. Both fractions were centrifuged and resuspended in 1 ml TSB. Cells were sonicated using 15 pulses, 500 msec, 50% power and OD_600_ was adjusted to 3. Cells were plated on TSA with 0.1 µg/ml rifampicin to estimate the number of spontaneous rifampicin-resistant mutants in the two fractions. Furthermore, cells were plated on TSA to estimate total CFU in the two fractions and mutation frequency was calculated as the number of rifampicin-resistant mutants per total CFU.

### MIC Determination

Cells were inoculated in TSB with added antibiotics to an OD_600_ of 0.01 and incubated at 37°C. The assay was performed in 96 well microtiter plates (100 µl culture per well). The MIC was defined as the lowest concentration from 2-fold dilution series of a given antibiotic that prevented growth. The following MICs were estimated: cefuroxime (CXM) = 4 µg/ml, erythromycin (ERM) = 0.25 µg/ml, rifampicin (RIF) = 0.032 µg/ml, vancomycin (VAN) = 2 µg/ml, kanamycin (KAN) = 0.8 µg/ml and ciprofloxacin (CIP) = 1 µg/ml. MIC did not vary between the strains used in the experiments (data not shown).

### Susceptibility to Antibiotics

Cells from two 8325-4 derivatives resistant to chloramphenicol and rifampicin, respectively were cultivated using standard growth conditions until visible cellular aggregates occurred in both (approx. 4 h). Six ml aggregated culture was separated into aggregate and dispersed cell fractions. Both fractions were washed in TSB and the aggregate fraction from one strain was mixed (1∶1) with the dispersed cell fraction from the other strain pre-adjusted to OD_600_ = 0.1. Cultures were re-incubated until OD_600_ = 0.5 when 25 × MIC of the antibiotics kanamycin (KAN), ciprofloxacin (CIP), erythromycin (ERM) and vancomycin (VAN) was added. Cultures were harvested after 0 h (control), 3 h (KAN and CIP) or 18 h (ERM and VAN) of incubation with antibiotics, depending on the killing kinetics of the antibiotic applied. To harvest the cells, cultures were separated in aggregated and dispersed cell fractions, centrifuged (6,300 G, 5 min) and resuspended in 1 ml 0.9% NaCl. Cells were then sonicated (15 pulses, 500 msec, 50% power) and plated on TSA; TSA with 10 µg/ml chloramphenicol or TSA with 0.1 µg/ml rifampicin to estimate total CFU, CFU of cells originating from the dispersed cell fraction and CFU originating from the aggregate fraction, respectively. Exponential cultures of the two strains included in the study were equally sensitive to the used antibiotics and had same growth rate (data not shown).

### Statistical Analysis

Averages were compared using two-tailed student’s t-test.

## Supporting Information

Figure S1 **Aggregates are held together by strong forces.** Aggregates from a post-exponential (OD_600_ = 2) culture of strain 8325-4 were analyzed using LDA. The aggregates were subjected to increasing shear forces (from shear 1 to shear 5) represented by increasing stirrer and pump speed. Error indicate standard deviation (n = 5)
(TIF)

Figure S2 **Aggregation of 8325-4 derivatives.** Post-exponential cultures of 8325-4 derivative strains were investigated by LDA for their ability to aggregate. Error bars indicate standard deviation (n = 5)
(TIF)

Figure S3 **Metabolic activity in biofilm and** ***ica*** **mutants.** Metabolic activity was determined and normalized to mg dry weight in (A): an overnight culture of 8325-4 by measuring reduced XTT (arbitrary units) of dispersed cells and dislodged biofilm or (B): dispersed and aggregated cells of wild type 8325-4 and 15981 as well as their isogenic *ica* mutants. Error bars indicate standard deviation (n = 3).
(TIF)

Figure S4 **Mutation frequency is increased in aggregating cells.** The aggregate fraction of a post-exponential 8325-4 culture was separated from the dispersed fraction. Mutation frequency was calculated as the number of rifampicin resistant mutants per total CFU. Error bars indicate standard deviation (n = 3).
(TIF)

Figure S5 **Quickspin removes most aggregates from supernatant.** A post-exponential culture of 8325-4 containing visible aggregates was subjected to the quick spin procedure (centrifugation 1400 rpm, 15s) and the supernatant was analyzed using LDA. All aggregates >50 µm were removed and the majority of the cells were present in the 1–15 µm size range. Error bars represent standard deviation (n = 5).
(TIF)

Text S1 **Supporting information, materials and methods.** Description of the LDA method and immune-detection method.
(DOCX)
