# Correction: Planktonic Aggregates of *Staphylococcus aureus* Protect against Common Antibiotics

**DOI:** 10.1371/annotation/08d0f2a8-0c40-4a0c-b546-0025648e73f0

**Published:** 2012-10-16

**Authors:** Jakob Haaber, Marianne Thorup Cohn, Dorte Frees, Thorbjørn Joest Andersen, Hanne Ingmer

The first two authors, Jakob Haaber and Marianne Thorup Cohn, should not have been attributed equal contribution. Jakob Haaber is the sole first author of this work. 

