# Supplementary figures and images for: Planktonic Aggregates of Staphylococcus aureus Protect against Common Antibiotics
Source: PLoS One. 2012 Jul 18;7(7):e41075. doi: 10.1371/journal.pone.0041075 (PMC3399816; doi:10.1371/journal.pone.0041075)

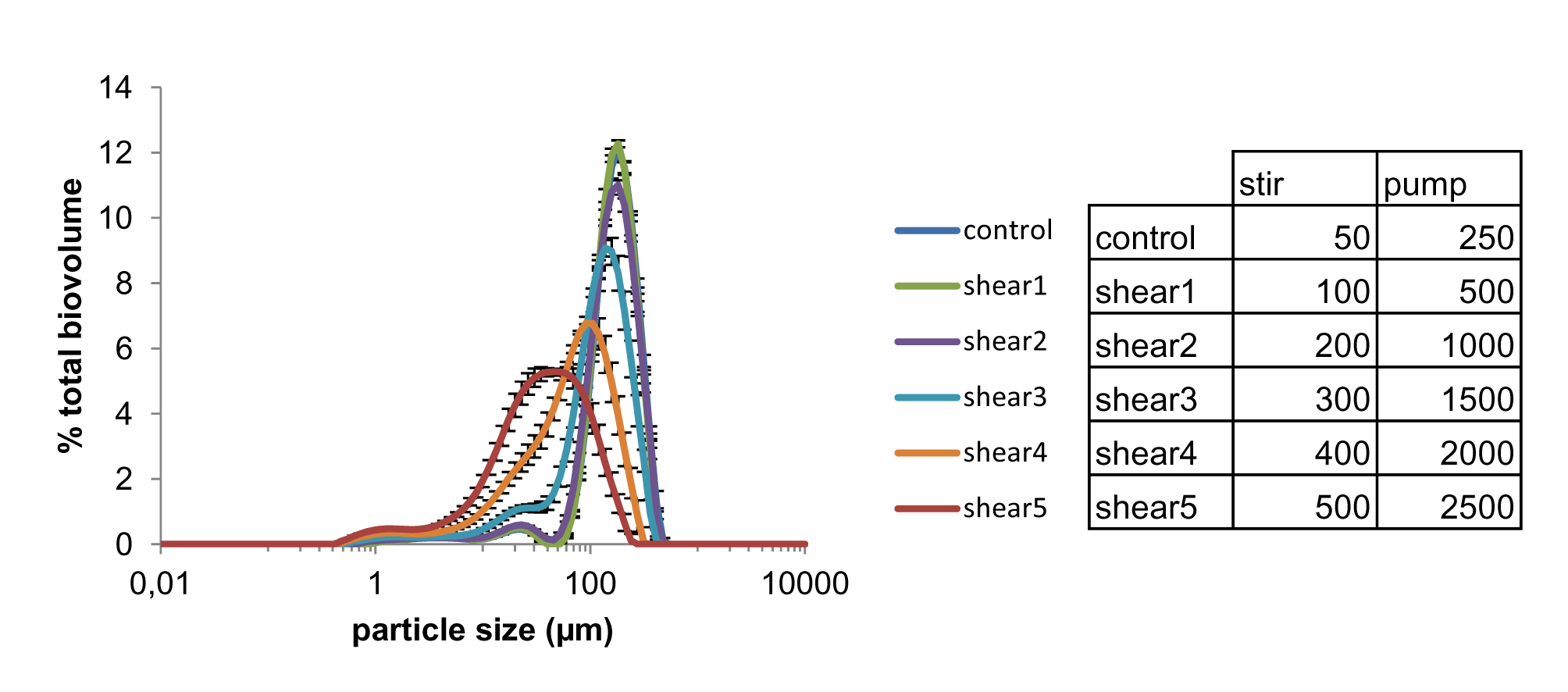

Supplement: Figure S1 — Aggregates are held together by strong forces. Aggregates from a post-exponential (OD600 = 2) culture of strain 8325-4 were analyzed using LDA. The aggregates were subjected to increasing shear forces (from shear 1 to shear 5) represented by increasing stirrer and pump speed. Error indicate standard deviation (n = 5) (TIF) [file pone.0041075.s001.tif]

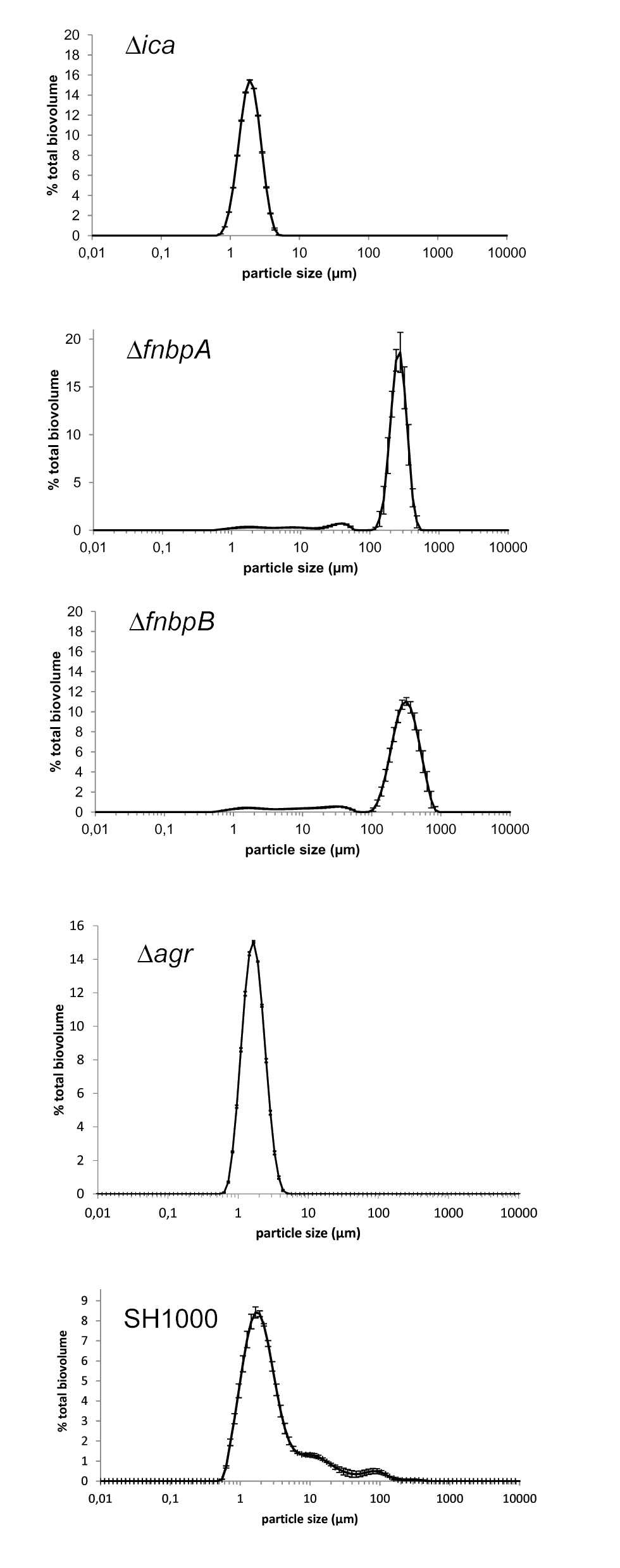

Supplement: Figure S2 — Aggregation of 8325-4 derivatives. Post-exponential cultures of 8325-4 derivative strains were investigated by LDA for their ability to aggregate. Error bars indicate standard deviation (n = 5) (TIF) [file pone.0041075.s002.tif]

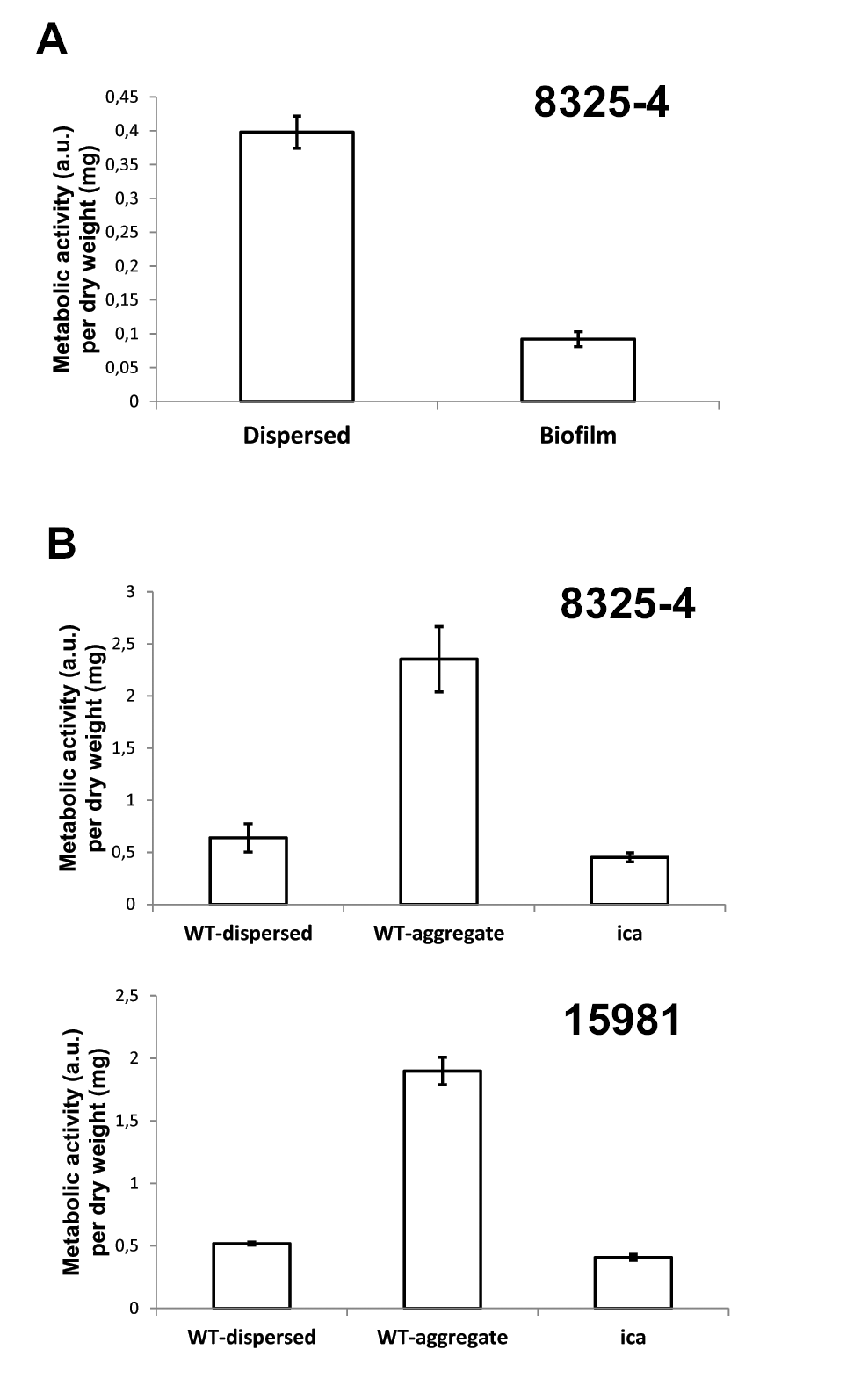

Supplement: Figure S3 — Metabolic activity in biofilm and ica mutants. Metabolic activity was determined and normalized to mg dry weight in (A): an overnight culture of 8325-4 by measuring reduced XTT (arbitrary units) of dispersed cells and dislodged biofilm or (B): dispersed and aggregated cells of wild type 8325-4 and 15981 as well as their isogenic ica mutants. Error bars indicate standard deviation (n = 3). (TIF) [file pone.0041075.s003.tif]

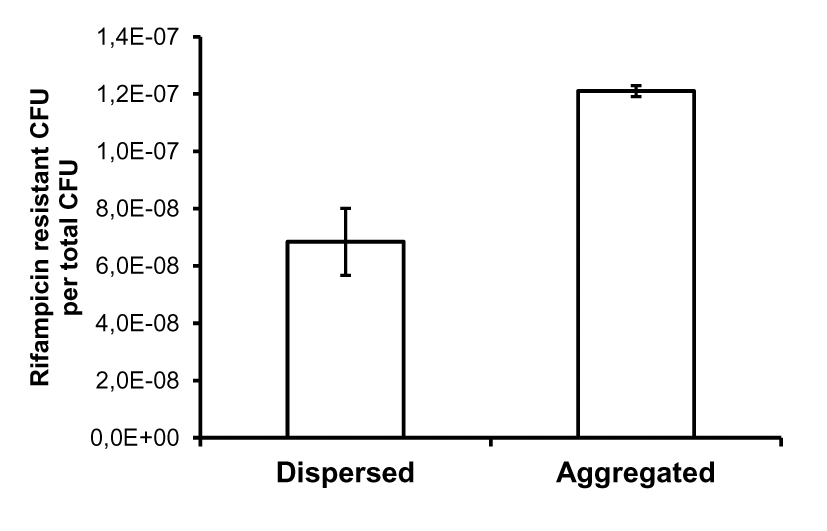

Supplement: Figure S4 — Mutation frequency is increased in aggregating cells. The aggregate fraction of a post-exponential 8325-4 culture was separated from the dispersed fraction. Mutation frequency was calculated as the number of rifampicin resistant mutants per total CFU. Error bars indicate standard deviation (n = 3). (TIF) [file pone.0041075.s004.tif]

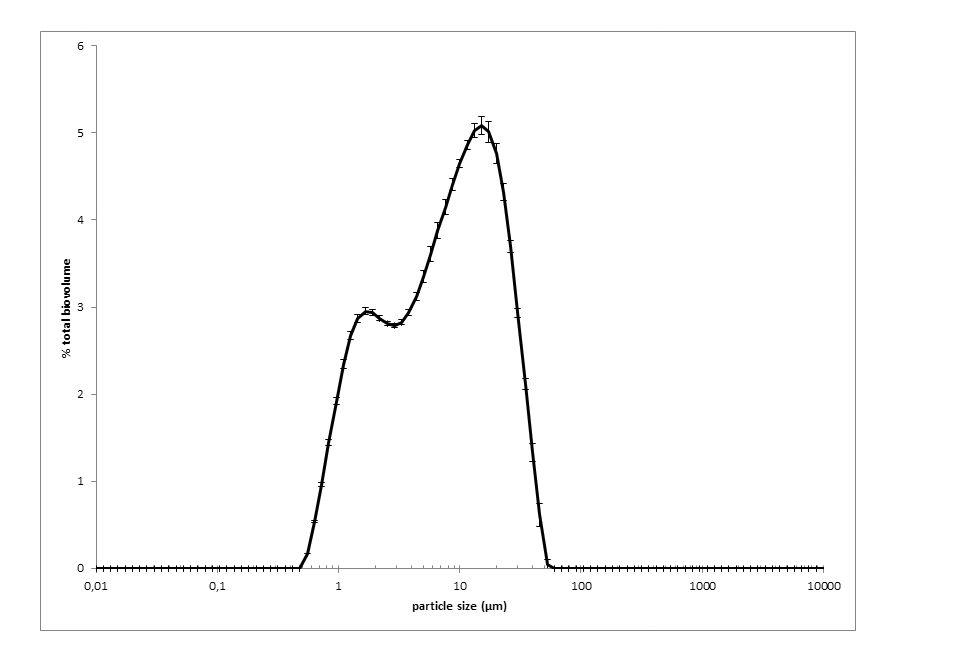

Supplement: Figure S5 — Quickspin removes most aggregates from supernatant. A post-exponential culture of 8325-4 containing visible aggregates was subjected to the quick spin procedure (centrifugation 1400 rpm, 15s) and the supernatant was analyzed using LDA. All aggregates >50 µm were removed and the majority of the cells were present in the 1–15 µm size range. Error bars represent standard deviation (n = 5). (TIF) [file pone.0041075.s005.tif]
