# Supplementary material for: Planktonic Aggregates of Staphylococcus aureus Protect against Common Antibiotics
Source: PLoS One. 2012 Jul 18;7(7):e41075. doi: 10.1371/journal.pone.0041075 (PMC3399816; doi:10.1371/journal.pone.0041075)
Supplement: Text S1 — Supporting information, materials and methods. Description of the LDA method and immune-detection method. (DOCX) [file pone.0041075.s006.docx]

**Supportive information: materials and methods:**

**LDA-particle sizing of aggregates in liquid cultures**

Measurements were performed using a Mastersizer 2000 (Malvern Instruments, Worcestershire, UK) in a size distribution range of 0.02-2000 µm divided into 89 size classes assuming spherical particles and applying the Mie theory to calculate particle sizes. The settings for dispersion medium and particles were “water” and “china clay”, respectively. The “china clay” setting is suitable for most irregularly shaped materials such as bacterial aggregates. As our standard condition we used a low stirring speed of 50 rpm and pump speed of 250 rpm, which kept aggregates in suspension and ensured an even flow of aggregates into the detection chamber while still keeping shear forces at a minimum. 20 mL of bacterial culture was added to the stir-tank and measurements were completed within 1 min. Re-measuring after prolonged intervals of stirring (5 min) did not change the size distribution thus demonstrating that the aggregates were not disintegrating due to shear effects in the system. The size-distributions are expressed in the unit [% bio-volume], which is calculated as the fraction of the volumetric contribution of particles within a given size class in relation to the total volumetric contribution of particles in all size classes. As the cell populations of both of the non-aggregating strains (Newman and SA564) were in the size fraction <6 µm, we used this value as the cut-off to distinguish between dispersed cell and aggregate fractions.

**Immunodetection analysis of PIA level**

Cells were grown using standard culture conditions and harvested either at OD_600_ of 2 or if 1/25×MIC was added to cultures when visible aggregation occurred (OD_600_ of 1-2). Cultures were centrifuged and pellet stored at -80°C. The pellet was resuspended in TSB to OD_600_ of 1 before 0,5M EDTA, pH 8.0 was added, and the sample was incubated at 95°C for 10 min. Cells were centrifuged and treated with proteinase K (20 mg/ml; Sigma) for 30 min at 37°C. 10μl of Tris-buffered saline (20mM Tris-HCl, 150mM NaCl, pH 7.4) containing 0.01 bromphenol blue was added and 5 μl spots were transferred to nitrocellulose filter (NuPAGE®, Invitrogen) and dried. The spots were blocked overnight with 5% skimmed milk in PBS with 0.1% TWEEN 20. Next day the membrane was washed twice in water and incubated 2 h with primary antibody raised against *S. aureus* PIA diluted 1:1000. Bound antibody was detected using peroxidase-conjugated goat anti-rabbit (IgG) antibody diluted 1:1000 (Amersham ECL Western Breeze® Immunodetection Kit, Invitrogen).
